# Psychotherapists' Readiness to Treat Refugee Patients and the Influence of Professional Quality of Life: A Cross‐Sectional Vignette Study

**DOI:** 10.1002/cpp.70076

**Published:** 2025-04-29

**Authors:** Pia Maria Schwegler, Theresa Neumann, Rita Rosner, Katharina Gossmann

**Affiliations:** ^1^ Department of Psychology Catholic University of Eichstätt‐Ingolstadt Eichstaett Germany

**Keywords:** professional quality of life, psychotherapy with refugees, refugee background, treatment readiness

## Abstract

**Background:**

Previous research has shown that psychotherapists' characteristics influence their readiness to treat refugee patients. The impact of therapists' professional quality of life (ProQOL) regarding their treatment readiness for refugee patients is unknown.

**Objective:**

This study aims to evaluate the ProQOL among psychotherapists in Germany. It examines how these factors and previous experience working with refugees affect psychotherapists' treatment readiness for refugee patients with symptoms of post‐traumatic stress disorder (PTSD).

**Method:**

In our study, we assessed the treatment readiness of licensed psychotherapists (LPTs) and psychotherapists in training (PiTs) using randomized refugee verus nonrefugee vignettes. Participants (*N* = 821) rated their treatment readiness for the presented case, reported their professional quality of life on the ProQOL questionnaire as well as prior experience with psychotherapy for refugees.

**Results:**

The ProQOL differed significantly between therapists with and without experience treating refugees: Compassion satisfaction was higher, and burnout lowers for those who had already treated refugees. Overall, treatment readiness was lower for refugee than for nonrefugee patients. Therapists with prior experience of working with refugees reported a significantly higher treatment readiness for the refugee vignette. Treatment readiness was not affected by the reported ProQOL.

**Conclusions:**

Treatment experience with refugees did not negatively impact therapists' ProQOL but fostered their further treatment readiness for this specific patient group. Psychotherapists should be encouraged to gain initial treatment experience with refugees to improve long‐term health care for refugees. Encouragement could be achieved by providing supervision or specialist training.

**Summary**

- Access to psychotherapy is particularly difficult for refugees in Germany, due to political, structural and individual factors, including psychotherapists' reservations.
- Psychotherapists' previous experience with refugees leads to higher compassion satisfaction, lower burnout and greater readiness to work with refugees again.
- Professional quality of life does not influence the readiness of psychotherapists to treat refugees with PTSD.
- However, it appears that psychotherapists in general are less willing to treat refugees than nonrefugees.
- In order to provide better psychotherapeutic care for refugees, it is important to address structural and bureaucratic barriers as well as individual barriers such as lack of supervision, self‐doubt and lack of knowledge.

**Abbreviations**
BO: burnout
CS: compassion satisfaction
LPT: licensed psychotherapist
PiT: psychotherapist in training
ProQOL: professional quality of life
STS: secondary traumatic stress

## 1 Introduction

In October 2024, the United Nations High Commissioner for Refugees reported 37.9 million refugees worldwide (UNHCR 2024). Of these, 65% originated from four countries: Syria, Venezuela, Ukraine and Afghanistan. A refugee is a person who has fled his or her home country due to a well‐founded fear of being persecuted for reasons of race, religion, nationality, membership of a particular social group or political opinion and is unable or unwilling, owing to such fear, to return (UNHCR 1951). So far in 2024, 179,212 asylum applications have been submitted in Germany (Bundesamt für Migration und Flüchtlinge 2024). Refugees have shown to be at higher risk to mental disorders due to the cumulative traumatic experiences associated with their displacement (Emmelkamp 2023; Blackmore et al. 2020; Schlaudt et al. 2020; Schmidt et al. 2023). According to the study by Nickerson et al. (2021), the most commonly reported experiences of refugees who fled to Australia were lack of food or water (44.2%), being close to death (43%) and experiencing torture (22.2%). Among Syrian refugees in high‐income Western countries, 40% reported symptoms of anxiety, 31% of depression and 31% of post‐traumatic stress disorder (PTSD) (Nguyen et al. 2022). Ukrainian refugees reported similar rates, with 51% experiencing anxiety symptoms and 44.7% experiencing depressive symptoms in Germany (Buchcik et al. 2023); 44.16% reported PTSD symptoms in a study from Portugal (Figueiredo et al. 2024).

Even after arriving in the host country, refugees often face multiple stressors (Emmelkamp 2023). Post‐migration stressors, such as discrimination in the host country, have been shown to negatively affect refugees' mental health (Grabo and Leavey 2023). Poor living conditions and limited social support increased PTSD and depression symptoms among refugees in Germany (Schilz et al. 2023). Language barriers, family concerns and anxiety about the asylum process also had a negative impact on mental health, whereas being treated fairly in the community, having enough food, financial support and access to health care and education had a positive impact (von Haumeder et al. 2019).

Given the potential high need for psychological support in the refugee population, it is important to note that psychosocial interventions were shown to significantly reduce PTSD, depression and anxiety symptoms in refugees (Molendijk et al. 2024; Turrini et al. 2019). The results of the meta‐analysis by Molendijk et al. (2024) supported the effectiveness of psychological treatments for adults, adolescents and refugee children. Positive effects are maintained over follow‐up periods of at least 1 month (Turrini et al. 2019) and up to 6 months (Kip et al. 2020). In particular, cognitive behavioural therapy (CBT) and eye movement desensitization and reprocessing therapy (EMDR) were shown to be effective in reducing symptoms of PTSD in refugees (Turrini et al. 2021). While Antuña‐Camblor and Hernandez (2025) reported no significant effects of EMDR on PTSD, Molendijk et al. (2024) found no differences in the methods studied but reported an overall effectiveness. Reports from refugee patients vary. Duden et al. (2020) found distrust of psychotherapy and concerns about stigma. Talking about problems was seen as both helpful and inappropriate. Trauma exposure in therapy gave meaning to experiences but was also stressful and worsening for some. While some found psychotherapy beneficial, others saw no improvement and preferred physical therapy or medication.

Although the need for and effectiveness of psychotherapy for refugees is high, accessing health care remains especially difficult for this population (BAfF e. V. 2024; Dumke, Wilker, et al. 2024). During the asylum process, health care costs are covered by the social welfare office (BAfF e. V. 2024). Within the first 18 months after arrival in Germany, it is difficult for refugees to get psychotherapy, as they are only entitled to medical care for acute illnesses or pain. Applications for psychotherapy and coverage are considered time‐consuming and unlikely to be successful. After 18 months, refugees are entitled to the same benefits as those with statutory health insurance. In addition, due to limited treatment capacity in Germany, there are long waiting lists for psychotherapeutic treatment (BAfF e.V. 2024). Other barriers include uncertain asylum status (Duden et al. 2020) and difficulties navigating a foreign health care system (Hahn et al. 2020). For refugee patients, a different understanding of mental health, fear of stigmatization and lack of awareness of available services can be a hindrance (Dumke, Wilker, et al. 2024). For psychotherapy in particular, language barriers, lack of trained translators and lack of clarity about funding are prominent barriers among psychotherapists (Duden et al. 2020; Dumke et al. 2023; Kiselev et al. 2020). When treatment was available, it was on average 20% shorter than for nonrefugee patients (Dumke et al. 2023). Only 26.1% of the refugees in need of treatment had access to mental health services 1 year after their arrival in Germany, with only 17.4% receiving minimally adequate treatment, and 4.3% receiving minimally adequate psychotherapy (Dumke, Schmidt, et al. 2024).

Furthermore, psychotherapists in Germany showed more therapy‐hindering attitudes and expected more negative emotions for themselves when treating refugees compared to nonrefugees (Dumke and Neuner 2022). As a result, they were generally less inclined to accept refugees as clients (Dumke and Neuner 2022; Schwegler et al. 2025). Factors perceived to influence therapists' treatment readiness include bureaucratic hurdles, organizational challenges, client motivation (Potter et al. 2023) and cultural differences, such as differing views on mental disorders and their treatment (Dumke, Wilker, et al. 2024; Peñuela‐O'Brien et al. 2023). Self‐doubt and comfort working with interpreters were identified as significant predictors for therapists' treatment readiness towards refugee patients (Schlechter et al. 2020). Prior experience with refugees, both private contact and in therapy, was shown to not only increase therapists' treatment readiness (Schlechter et al. 2020) but also reduce their therapy‐hindering attitudes towards refugee patients (Dumke and Neuner 2022).

The professional quality of life (ProQOL) of psychotherapists is another aspect to be considered in relation to their readiness to treat refugees. In general, working as a psychotherapist can involve a high level of responsibility, emotional strain, but also openness and tolerance (Råbu et al. 2016). Given the higher prevalence of PTSD among refugees (Nguyen et al. 2022; Figueiredo et al. 2024), there is evidence that working with forcibly displaced people can lead to increased emotional distress in the form of burnout (BO) and secondary traumatic stress (STS) among mental health care providers from different countries like Turkey, Greece or Iraq (Brooks et al. 2022; Ghafoori et al. 2024; Kizilhan 2020; Roberts et al. 2021). In addition to exposure to trauma reports, post‐migration factors such as resettlement difficulties and political and social barriers lead to frustration and disappointment among Australian caregivers (Puvimanasinghe et al. 2015). This had a cumulative effect on symptoms of secondary traumatization. Conversely, research has also shown that working with refugees is associated with an increase in compassion satisfaction (Posselt et al. 2019) and can have a positive and impowering impact on service providers in Australia (Posselt et al. 2019; Puvimanasinghe et al. 2015). In the interview study conducted by Puvimanasinghe et al. (2015), the majority of participants indicated that their motivation to engage with refugees was derived from their prior experience in this field. They reported that through their appreciation and awareness of the refugees' strengths and resilience, caregivers internalized these as positive experiences.

To summarize, various barriers exist in the psychotherapeutic treatment of refugees. In addition to bureaucratic aspects (BAfF e. V 2024), cultural differences (Potter et al. 2023; Peñuela‐O'Brien et al. 2023) and reservations among psychotherapists (Dumke and Neuner 2022; Schwegler et al. 2025), it is possible that the ProQOL of German psychotherapists has an influence on their readiness to treat refugees. Increased stress among psychotherapists could lead to a decreased readiness to treat refugees. However, there is a lack of research on the relationship between ProQOL, experience of treating refugees and readiness to treat refugees among German psychotherapists working in outpatient settings, including licensed therapists and those still in training. Therefore, the main aim of this study was to find out whether the ProQOL of German psychotherapists influenced their readiness to treat refugees. Furthermore, this study aimed to reflect the situation of outpatient psychotherapeutic care in Germany as realistically as possible. The survey was designed to be as broad as possible and included both experienced psychotherapists and psychotherapists in training (PiTs), different specializations (e.g., treating adults or children and adolescents, working with behavioural therapy or depth psychology) and different working contexts (self‐employed or employed in a clinic). In addition, we aimed to replicate the results of Schlechter et al. (2020) using patients' vignettes on a larger sample. A further aim was to find out whether the readiness to treat refugees was influenced by previous treatment of refugees. In summary, the following hypotheses were proposed: (1) Previous experiences in working with refugees has an impact on the ProQOL (BO, STS and CS) of German psychotherapists; (2) the ProQOL (BO, STS and CS) of German psychotherapists influences their readiness to treat refugees with PTSD symptoms; (3) previous experience in treating refugees will lead to a higher readiness to treat refugees.

## 2 Methods

### 2.1 Ethics

The university's ethics board approved the study (approval number: 121‐2022). Before participating in the online survey, all participants were informed about the aims and duration of the study, the anonymous design, the applicable data protection regulations and the participation requirements. We did not include information about the case vignettes in the study invitation as the survey was designed as an experimental study with randomly presented case vignettes. To participate, interested psychotherapists had to give their informed consent.

### 2.2 Recruitment and Participants

The study design corresponds to a nationwide anonymous online survey with experimental aspects using randomly presented case vignettes. According to the participation requirements, only psychotherapists—either licensed (LPTs) or in training (PITs)—could participate. Psychotherapists were informed about the survey by different regional and national psychotherapist associations as well as psychotherapeutic training institutes who distributed the survey among their members via newsletters, journals, separate emails or internal cloud servers. Additionally, in accordance with the referrals of some federal associations for LPTs, we searched the publicly available directories of these associations for email addresses of LPTs to inform them about the survey. For the recruitment of PiTs, we compiled a directory of all training institutes for psychotherapy in Germany by federal state and therapeutic approach. From this directory, we randomly selected one institute in each federal state for each therapeutic approach, if available. After contacting the selected institutes and obtaining their agreement, the institutions distributed the survey to all their trainees. If an institute did not agree or answer, the next institute with the same therapeutic approach for the respective federal state was contacted.

The online survey took about 15 min and a total of *N* = 1032 LPTs and PiTs participated. Of these, we excluded *n* = 211 data sets from the statistical analysis due to dropping out before the case vignettes were presented or missing data related to demographics or ProQOL. The final sample included in the statistical analysis comprised *N* = 821 psychotherapists.

### 2.3 Procedure

**FIGURE 1:**
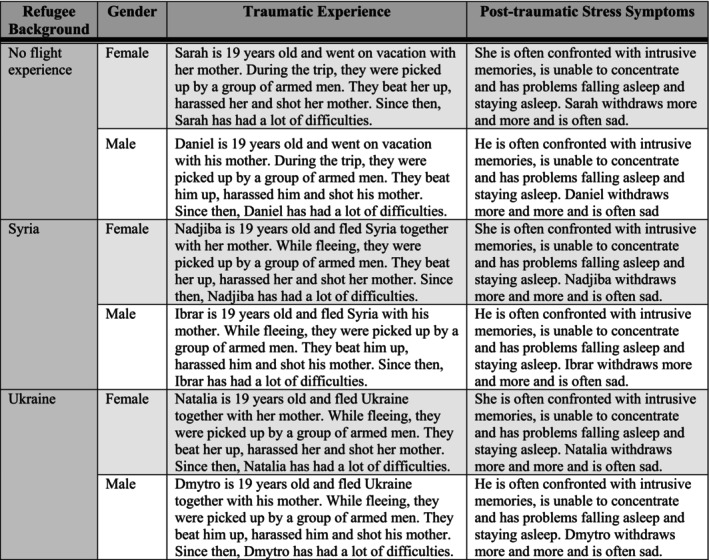
Randomized vignettes.

The study data were collected anonymousl!!!y with Qualtrics (Provo, UT, 2020) between October 2022 and August 2023. After providing informed consent, the psychotherapists shared demographic details such as their age, along with professional information, including their prior experience in providing psychotherapy to refugee patients. After the demographical section, the participants reported their ProQOL on the ProQOL questionnaire (Stamm 2010). Afterwards, one of six case vignettes was randomly presented: Each of them described a patient with PTSD symptoms with or without a refugee background (see Figure 1). The psychotherapists then rated their readiness to treat the portrayed patient. The survey was conducted entirely in German, and the psychotherapists participated voluntarily without receiving any incentives.

### 2.4 Material

#### 2.4.1 Measures

Participants provided sociodemographic and professional information during the survey, which can be found in Tables 1 and 2.

**Table 1:** Demographic information about participating LPTs and PiTs.

*Variable*	*N* = 821					
	*n*	M	SD	Mdn	Min	Max
Age (years)	821	45.44	12.21	44	24	80
Licensed for (years)	562	12.20	8.29	10	0	42
Own practice for (years)	460	10.95	9.11	8	0	50
Employed by a clinic/hospital for (years)	93	8.90	7.89	7	1	32
Number of therapy sessions per week	821	18.76	8.94	20	0	50
Number of PTSD patients treated	797	47.83	218.71	15	0	5400
Number of refugee patients treated	395	11.97	27.34	5	0	250
Number of refugee patients with PTSD treated	395	8.32	17.94	3	0	150

**Table 2:** Demographic information about participating LPTs and PiTs.

Variable	*N* = 821	
	*n*	%
Training status		
Completed training	590	71.9
Currently in training	203	24.7
Diploma or master's degree without training	24	2.9
Other	4	0.5
Type of licence		
Psychotherapist for children and adolescents	154	18.8
Psychological psychotherapist	350	42.6
Psychological psychotherapist also licensed for treating children and adolescents	65	7.9
Medical psychotherapist	17	2.1
Other	5	0.6
Employed by a clinic/hospital	93	11.7
Own practice	472	59.5
Approved for public health care insurance	473	59.6
Specific trauma training^a^		
EMDR	316	38.5
TF‐CBT	319	38.9
NET	235	28.6
Imaginary rescripting	232	28.3
Hypnotherapy	140	17.1
CPT	88	10.7
Play therapy	80	9.7
Somatic experiencing	36	4.4
Other	168	20.5
None	131	16.0
Gender		
Female	687	83.7
Male	129	15.7
Diverse	5	0.6
Working with refugees		
Yes	399	48.6
Migration background^a^		
Yes, myself	38	4.9
Yes, my parents	61	7.4
Flight experience		
Yes	8	1.0
Therapeutic approach^a^		
Behavioural therapy	623	75.9
Psychodynamic approach	213	25.9
Systemic	48	5.8
Other	31	3.8

Abbreviations: CPT, cognitive processing therapy; EMDR, eye movement desensitization and reprocessing; NET, narrative exposition therapy; PiT, psychotherapist in training; LPT, licensed psychotherapist; TF‐CBT, trauma‐focused cognitive behavioural therapy.

^a^ Multiple answers possible.

Therapists were randomly assigned one of six case vignettes (see below) and asked to rate their treatment readiness for the patient described in the vignette using a single‐item visual analogue scale (VAS) ranging from 0 (*Low*) to 100 (*High*): ‘How willing are you to treat the described person psychotherapeutically?’

#### 2.4.2 Case Vignettes

According to the experimental study design, we developed six parallel vignettes (65 to 66 words each), varying the patient's gender (female vs. male), refugee background (refugee vs. nonrefugee) and, for the two flight vignettes, their country of origin (Ukraine vs. Syria). The Ukraine versus Syria vignettes were parallelised by using an identical wording except for the names of the described persons as well as their country of origin. For a maximum parallelization between the refugee versus nonrefugee vignettes, the nonrefugee vignettes were designed with the same traumatic experience during a vacation as the refugee vignettes experienced during flight. All six vignettes are depicted in Figure 1.

#### 2.4.3 ProQOL

The ProQOL is a self‐assessment instrument designed to evaluate the impact of working with individuals who have coped with significant traumatic stress (Stamm 2010). It includes 30 items rated on a 5‐point Likert scale (1, *Never* to 5, *Very often*), equally divided into three subscales: compassion satisfaction (e.g., ‘My work makes me feel satisfied’), BO (e.g., ‘I feel worn out because of my work as a *[helper]*’) and STS (e.g., ‘I think that I might have been affected by the traumatic stress of those I *[help]*’). These subscales demonstrated acceptable to high internal consistency, with Cronbach's alpha values of α = 0.88, 0.75 and 0.81, respectively (Stamm 2010). Higher scores indicate greater levels of each dimension (range 10 to 50), with cut‐offs for low, moderate and high levels as specified for each scale.

### 2.5 Statistical Analyses

As the research question only distinguished between patients with and without a refugee background, the treatment readiness ratings for the Ukrainian and Syrian vignettes were combined for the analysis and compared to the ratings for the nonrefugee vignettes. First, we tested all statistical requirements for our statistical tests to be performed. To compare the ProQOL ratings between psychotherapists who had already treated refugee patients and those who had not, we then conducted a *t*‐test for independent samples for each of the ProQOL subscales. To analyse potential predictors for therapists' treatment readiness, a hierarchical multiple regression was performed with treatment readiness as the dependent variable. As a first block, we included the ProQOL subscales in our regression model. In addition, the vignette characteristics (refugee vs. nonrefugee) were added in a second block. The third block further included the refugee‐specific treatment experience of the therapists as a dichotomous variable (previous psychotherapy with refugees: yes vs. no) in interaction with the refugee background of the case vignette. The fourth block then also considered the interaction of each ProQOL subscale with the refugee background of the case vignette.

## 3 Results

### 3.1 Descriptive Statistics

The final data set consisted of *N* = 821 participants (*n* = 590 LPTs, *n* = 203 PiTs, *n* = 24 master's/diploma degree holders without licensure, *n* = 4 with other qualifications) with a mean age of *M* = 45.44 years (*SD* = 12.21, *Mdn* = 44). The vast majority of the participants were female (83.7%, *n* = 687). Five participants identified as gender diverse, 12.3% (*n* = 99) reported a migration background, with *n* = 8 having been refugees themselves. Additionally, *n* = 399 (48.6%) had prior experience working with refugees. Further information about sociodemographics can be found in Tables 1 and 2.

**FIGURE 2:**
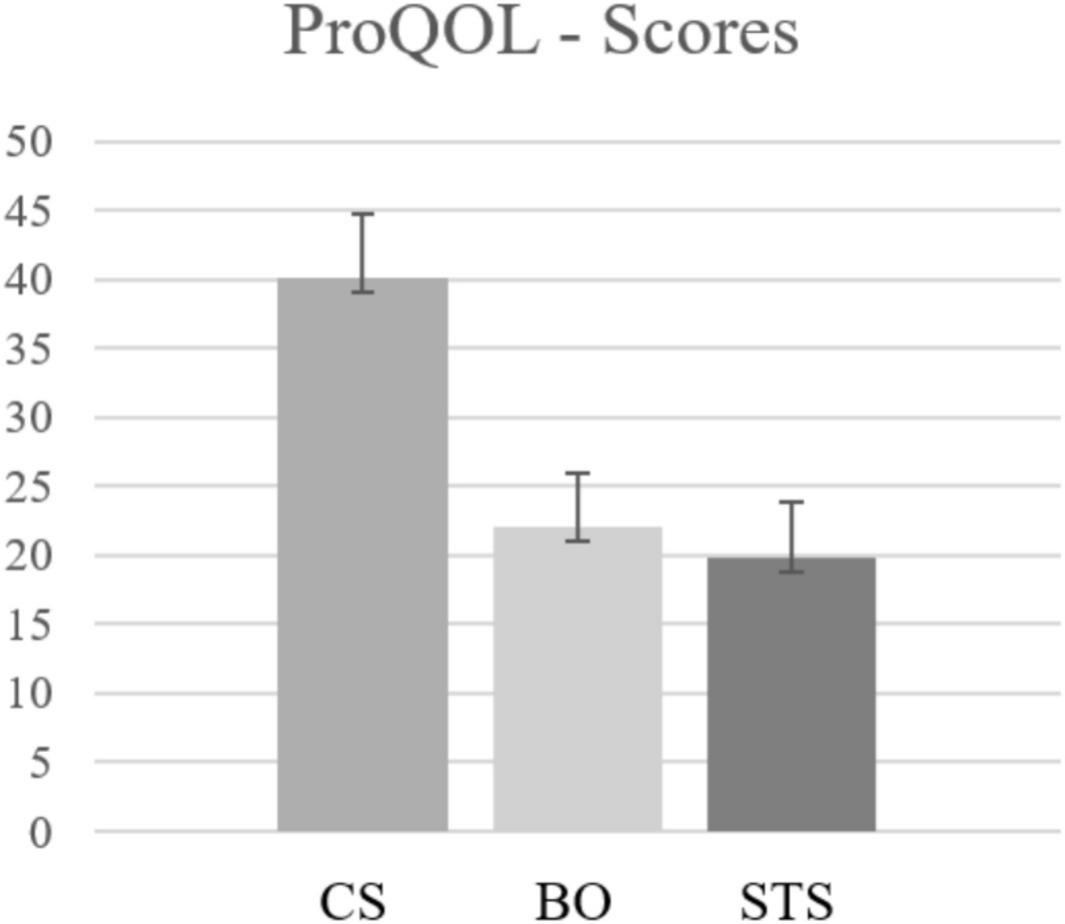
ProQOL‐Scores of the participating psychotherapists (BO, burnout; CS, compassion satisfaction; STS, secondary traumatic stress).

The distribution of the vignettes can be found in Table 3. The overall mean readiness to treat the vignette patient was *M* = 80.31 (*SD* = 22.65; *Mdn* = 87; *Min* = 0; *Max* = 100, *N* = 821), with 60.3% of the participants scoring above the mean. The ProQOL showed moderate CS levels (*M* = 40.14, *SD* = 4.60, *Mdn* = 40) and low BO (*M* = 22.02, *SD* = 3.97, *Mdn* = 22) and STS levels (*M* = 19.80, *SD* = 4.13, *Mdn* = 19). ProQOL‐Scores are shown in Figure 2.

**Table 3:** Distribution of the vignettes.

Vignettes	*N* = 821	
	*n*	%
Flight—Ukraine—male	143	17.4
Flight—Ukraine—female	143	17.4
Flight—Syria—male	135	16.4
Flight—Syria—female	140	17.1
No flight—Germany—male	141	17.2
No flight—Germany—female	119	14.5

On average, participants who had already worked with refugees reported more CS (*M* = 40.53, *SE* = 0.22) than those who had not (*M* = 39.77, *SE* = 0.22). This difference, 0.76, BCa 95% CI [0.131, 1.387], was significant [*t*(818.97) = 2.37, *p* = 0.009] and represented a small effect size, *d* = 0.17. Furthermore, therapists who had previously worked with refugees reported less BO on average (*M* = 21.71, *SE* = 0.19) than those who had not (*M* = 22.32, *SE* = 0.20). This difference, −0.60, BCa 95% CI [−1.147, −0.060], was significant as well [*t*(819) = −2.18, *p* = 0.015] with a small effect size, *d* = −0.15. There was no significant difference for STS.

### 3.2 Models to Predict Treatment Readiness

The results of the hierarchical regression analysis indicated that all four models were statistically significant (*p* < 0.001). Model 1, which included the three ProQOL scales as independent variables, explained 6.6% of the variance in the treatment readiness [*F*(3,817) = 20.43, *p* < 0.001, *n* = 821]. It was found that with an increase in CS, readiness also increased significantly by a factor of 1.05 (*β* = 0.21, *p* < 0.001). CS remained significant across all subsequent models. In contrast, with an increase in STS, readiness decreased significantly by a factor of −0.43 (*β* = −0.08, *p* = 0.047). STS did not prove to be a significant predictor in any of the following models.

Model 2 explained 9.3% of the variance in the treatment readiness [*F*(4,816) = 22.13, *p* < 0.001, *n* = 821]. The allocation of a flight vignette significantly lowered the therapists' treatment readiness by −8.16 (*β* = −0.17, *p* < 0.001). The negative correlation between flight vignette and treatment readiness remained significant in Models 3 and 4.

The inclusion of the interaction variable (previous work experience with refugees and flight vignette) as a predictor in Model 3 explained a further 3.2% of the variance [*F*(5,815) = 22.36, *p* < 0.001, *n* = 821]. It can be observed that participants who had previously worked with refugees and were assigned a flight vignette demonstrated an average increase in treatment readiness of 8.30 (*β* = 0.17, *p* < 0.001).

The final model accounted for 11.7% of the variance following the inclusion of the three interaction variables (ProQOL scales and flight vignette) [*F*(8,812) = 14.58, *p* < 0.001, *n* = 821]. The interactions of the three ProQOL subscales (BO, CS and STS) with the flight vignette did not contribute significantly to the final regression model. For more information, see Table 4.

**Table 4:** Multiple regression.

	Readiness to treat						
	*b*	*SE B*	*β*	*t*	*p*	*R* ^2^	*Adj. R* ^2^
Model 1						0.07	0.07
Constant	49.90 (25.47, 74.34)	12.45		4.01	< 0.001		
ProQOL Sumscore CS	1.05 (0.64, 1.46)	0.21	0.21	5.01	< 0.001*		
ProQOL Sumscore BO	−0.15 (−0.69, 0.39)	0.27	−0.03	−0.55	0.582		
ProQOL Sumscore STS	−0.43 (−0.85, −0.01)	0.22	−0.08	−1.99	0.047*		
Model 2						0.10	0.09
Constant	55.11 (30.95, 79.27)	12.31		4.48	< 0.001		
ProQOL Sumscore CS	1.05 (0.64, 1.46)	0.21	0.21	5.08	< 0.001*		
ProQOL Sumscore BO	−0.17 (−0.70, 0.36)	0.27	−0.03	−0.63	0.529		
ProQOL Sumscore STS	−0.39 (−0.80, 0.03)	0.21	−0.07	−1.82	0.068		
Flight vignette	−8.16 (−11.34, −4.98)	1.62	−0.17	−5.04	< 0.001*		
Model 3						0.12	0.12
Constant	54.74 (30.87, 78.61)	12.16		4.50	< 0.001		
ProQOL Sumscore CS	1.03 (0.62, 1.43)	0.21	0.21	5.02	< 0.001*		
ProQOL Sumscore BO	−0.12 (−0.64, 0.41)	0.27	−0.02	−0.43	0.665		
ProQOL Sumscore STS	−0.38 (−0.79, 0.03)	0.21	−0.07	−1.80	0.072		
Flight vignette	−12.34 (−15.95, −8.73)	1.84	−0.25	−6.71	< 0.001*		
Refugee Work × flight vignette (interaction variable)	8.30 (4.75, 11.84)	1.81	0.17	4.59	< 0.001*		
Model 4						0.13	0.12
Constant	40.84 (−0.52, 82.19)	21.07		1.94	0.053		
ProQOL Sumscore CS	1.02 (0.31, 1.73)	0.36	0.21	2.83	0.005*		
ProQOL Sumscore BO	0.26 (−0.64, 1.15)	0.46	0.05	0.57	0.572		
ProQOL Sumscore STS	−0.08 (−0.80, 0.65)	0.37	−0.01	−0.20	0.839		
Flight vignette	−12.28 (−15.89, −8.67)	1.84	−0.25	−6.68	< 0.001*		
Refugee work × flight vignette (interaction variable)	8.09 (4.54, 11.64)	1.81	0.17	4.47	< 0.001*		
ProQOL Sumscore BO × flight vignette (interaction variable, centred)	−0.56 (−1.66, 0.54)	0.56	−0.08	−1.00	0.319		
ProQOL Sumscore STS × flight vignette (interaction variable, centred)	−0.43 (−1.31, 0.45)	0.45	−0.07	−0.96	0.339		
ProQOL Sumscore CS × flight vignette (interaction variable, centred)	−0.01 (−0.87, 0.85)	0.44	−0.00	−0.02	0.982		

*Note: p*, significance, * highlights  the significant *p*‐values (*p ≤* 0.05).

Abbreviations: *b*, regression coefficient, confidence interval is shown in brackets; *β*, standardized coefficient beta; BO, burnout; CS, compassion satisfaction; ProQOL, professional quality of life; *SE B*, standardized error; STS, secondary traumatic stress; *t*, test statistics.

## 4 Discussion

The aim of this study was to find out whether the ProQOL of German psychotherapists influenced their readiness to treat refugees. We aimed to replicate the results of Schlechter et al. (2020) and to find out whether the readiness to treat refugees was influenced by previous treatment of refugees. We have hypothesized that (1) previous experiences in working with refugees has an impact on the ProQOL (BO, STS and CS) of German psychotherapists; (2) the ProQOL (BO, STS and CS) of German psychotherapists influences their readiness to treat refugees with PTSD symptoms; (3) previous experience in treating refugees will lead to a higher readiness to treat refugees.

In conclusion, the assessed sample had moderate CS scores and low BO and STS scores. Those participants who had previously worked with refugees reported elevated CS and diminished BO levels. Higher CS and lower STS were associated with greater readiness to treat vignette patients in general. The vignette experiment showed a significantly lower readiness of psychotherapists to treat patients with a refugee background. Participants who had previous experience of working with refugees and had received a flight vignette reported higher readiness to treat refugee patients. However, the ProQOL had no impact on treatment readiness regarding refugees.

Based on the results of the ProQOL, the ProQOL among the psychotherapists in this study was generally favourable as CS was moderate on average, while BO and STS were small. It was found that previous experience in psychotherapy with refugees does not lead to higher STS scores among practitioners. This is contrary to previous research (Brooks et al. 2022; Ghafoori et al. 2024; Kizilhan 2020; Roberts et al. 2021). They even reported more CS, like in the study of Posselt et al. (2019), and less BO than those who had no experience with psychotherapy with refugees. These differences in CS and BO might be based on special interests and engagement of psychotherapists. It is possible that psychotherapists with a high devotion to psychotherapy with refugees feel more compassion towards this population. Watching refugee patients gain independence and confidence as they overcome systematic barriers and move forward in life can empower service providers by reinforcing the effectiveness and value of their services (Puvimanasinghe et al. 2015). Given that BO symptoms may occur due to coping difficulties (Ghafoori et al. 2024), it is possible that our sample learned self‐care strategies or attended training to prevent BO and STS (Hernández et al. 2010). Brooks et al. (2022) linked lower organizational support to higher STS scores and lower social support to anxiety among service providers working with Syrian refugees. Therefore, it is possible that there is a stronger support network in our sample that was not captured. Further research should focus more on the possible reasons for the difference in BO between psychotherapists working with refugees and those without such experiences. Knowledge and strategies for BO prevention, long hours, demanding workload (Ghafoori et al. 2024) and valence of therapy experiences with PTSD patients with and without a refugee background should be considered as potential predictors for BO and ProQOL in general. Additionally, psychotherapists with a low quality of life might not have participated in the study due to their own distress and a lack of time. Thus, more comprehensive assessments are needed to prevent biases due to self‐selection. For example, incentives could be used to motivate psychotherapists with a lower ProQOL to still participate in such a study.

When considering the influence of ProQOL on treatment readiness for the described vignette patients, CS was related positively and STS negatively to treatment readiness. Higher STS might reduce readiness to treat PTSD patients, as STS may cause clinicians to avoid activities that remind them of the trauma (Stamm 2010). However, the influence of STS on treatment readiness disappeared when considering the refugee background of the vignette. In contrast, CS seemed to be a more stable predictor for the readiness to treat a patient with PTSD symptoms, regardless of their refugee background. Duden et al. (2020) reported positive effects of working with refugees, including finding meaning in their work. It is possible that this meaning lies in our participants' stable compassion satisfaction. Nevertheless, a supportive network and specialist supervision are essential for the ProQOL of psychotherapists working with traumatized refugees (Duden et al. 2020; Peñuela‐O'Brien et al. 2023; Puvimanasinghe et al. 2015).

Our study demonstrated that the treatment readiness towards PTSD patients with a refugee background was lower than towards PTSD patients with the same traumatic event but without a refugee background. Not the symptoms of PTSD but only the refugee status was relevant for treatment readiness. This is consistent with the findings of Dumke and Neuner (2022) and Schwegler et al. (2025). The facets of ProQOL did not influence the readiness to treat the refugee vignette. Accordingly, CS as an aspect of ProQOL positively influenced the readiness to treat PTSD patients, but not the readiness to treat refugees with PTSD. Therefore, further research needs to explore potential predictors for the treatment readiness towards refugee patients beyond the ProQOL including BO, CS and STS. Factors affecting the treatment readiness towards refugees might be attitudes towards refugees in general (Dumke and Neuner 2022; Schwegler et al. 2025), lack of language skills or personal fears and doubts (Schlechter et al. 2020). At the time of the survey, 24.7% of the participants were in training. This could mean that self‐doubt and a lower level of experience and confidence in treating refugees with PTSD may lead to a lower level of readiness. On the other hand, PiTs have a lower caseload and receive closer supervision, which should be used to encourage them to treat refugees. Removing barriers at the structural level can be achieved, for example, through improved cooperation between institutions to reduce the burden on psychotherapists regarding refugees' needs beyond their scope of practice (Peñuela‐O'Brien et al. 2023). Another aspect is simplifying the process of securing funding for treatment and interpreters (Duden et al. 2020).

When considering therapists' experiences with psychotherapy with refugees next to their ProQOL and the patients' refugee status, the effects of therapists' CS and patients' refugee background remained stable. There was an additional interaction between the vignette patient's refugee background and therapists' previous experiences with psychotherapy with refugees. If psychotherapists had already treated refugees, they reported a higher treatment readiness for refugee patients than those who had not. This could be attributed to the aforementioned empowerment (Duden et al. 2020) and meaningfulness of working with refugees (Puvimanasinghe et al. 2015). The results are consistent with the findings of Schlechter et al. (2020) and have been successfully replicated. The lower readiness to treat refugee patients in comparison to nonrefugee patients may be attributed to the presence of participants in the sample who have no prior experience working with refugees or treating PTSD. Thus, fostering first treatment experiences with a refugee patient, for example, during psychotherapy training, might enhance therapists' readiness to treat further refugee patients. Special training and information on psychotherapy with refugees is needed to avoid helplessness and loss of confidence (Peñuela‐O'Brien et al. 2023). Additionally, further research should also consider that the interaction between a patient's refugee background and psychotherapeutic experiences with refugees could be moderated by a third variable. The already mentioned therapists' attitudes towards refugees or their trauma‐specific training could act as such moderating variables and could be examined in studies including special training trials and control groups.

### 4.1 Limitations

For the recruitment of participants, we used a random selection of psychotherapeutic associations and training institutes. All members of these associations and institutions with available contact information had the opportunity to see the study invitation. However, participation was voluntary and thus self‐selection is likely. It is likely that more psychotherapists with a special interest in treating PTSD participated in the study. This is also a possible explanation for the generally high treatment readiness among the participants. Therefore, an overestimation of the level of treatment readiness based on a self‐selection bias is possible. Conversely, therapists with higher BO and STS scores may have been unable to participate due to a lack of capacity to complete additional surveys. Additionally, the high level of treatment readiness in this study could be based on social desirability. The anonymous study design and the experimental case vignettes were used as strategies to reduce potential effects of social desirability. In order to include as many psychotherapists as possible, we set the age of the patient in the vignette to 19 years, allowing both adult and child and adolescent therapists to participate. Some may have reservations or preferences for this transitional age group, which may have influenced treatment readiness. Further subdivision of the vignettes would have weakened the statistical power. However, the heterogeneous sample should be taken into account when interpreting the results.

At the time of recruitment, there was increased media coverage of the invasion of Ukraine and the resulting displacement of people. As the media can also play an important role in shaping public attitudes towards refugees (Kosho 2016; Tsai et al. 2023), this could have an impact on the treatment readiness that we did not capture and thus should be considered in the interpretation of the results.

Health care systems vary among different countries and thus, the results of this study may not be fully generalizable to psychotherapists in other health care systems. Even though we assessed the refugee background of the participants, the number of psychotherapists with such a background was very small (*n* = 8; 1.0%). Due to this lack of heterogeneity in cultural backgrounds, we could not consider the own refugee background of the psychotherapists as a correlate in the analyses. This might limit the generalizability of the results for countries with more cultural heterogeneity among psychotherapists.

## 5 Conclusions

This study aimed to assess the ProQOL among psychotherapists in Germany and relate this to their treatment readiness towards PTSD patients with and without a refugee background. Moreover, therapists' previous experiences with refugee patients were also considered. Even though our results demonstrated that the ProQOL was favourable on average for our participants, CS and BO differed depending on previous psychotherapy experience with refugees.

Regarding treatment readiness for PTSD in total, only CS proved to be a stable positive predictor. None of the ProQOL subscales influenced the treatment readiness towards refugee patients. Therapists' treatment readiness was lower for patients with a refugee background than for those without a refugee background, when controlling symptoms and traumatic experiences. However, when psychotherapists reported previous treatment of refugees, they had a higher average treatment readiness for the described refugee patients. Our results on treatment readiness need to be considered when discussing the improvement of psychotherapeutic treatment for PTDS patients and refugee patients with PTSD. Interventions to encourage first treatment experiences with refugees and to improve CS of psychotherapists seem to be relevant. Therefore, trauma‐specific training also with regard to refugee patients as well as supervision could be helpful. This might increase the treatment readiness for PTSD and refugee patients among psychotherapists and thus improve psychotherapeutic care for this population.

## Author Contributions

Pia Maria Schwegler and Katharina Gossmann designed the study and prepared the manuscript draft. Pia Maria Schwegler, Katharina Gossmann and Theresa Neumann recruited participants and collected data, with Pia Maria Schwegler conducting the statistical analysis. Katharina Gossmann and Rita Rosner supervised the study, while Katharina Gossmann also provided guidance on the statistical analysis and interpretation of the results. All authors reviewed, revised and approved the final version of the manuscript.

## Ethics Statement

The study was approved by the Institutional Review Board of the Catholic University of Eichstätt‐Ingolstadt in December 2022 (Ethics Approval Number: 121‐2022).

## Consent

All participants provided written informed consent to take part in the study.

## Conflicts of Interest

The authors declare no conflicts of interest.

## Data Availability

The generated and analysed data along with the statistical code used for the analyses are available on request from the corresponding author. These materials are not publicly accessible due to privacy considerations and ongoing analyses.
